# Isolation of Chitin from Black Soldier Fly (*Hermetia illucens*) and Its Usage to Metal Sorption

**DOI:** 10.3390/polym13050818

**Published:** 2021-03-07

**Authors:** Katarzyna Złotko, Adam Waśko, Daniel M. Kamiński, Iwona Budziak-Wieczorek, Piotr Bulak, Andrzej Bieganowski

**Affiliations:** 1 Institute of Agrophysics, Polish Academy of Sciences, Doświadczalna 4, 20-290 Lublin, Poland; k.zlotko@ipan.lublin.pl (K.Z.); p.bulak@ipan.lublin.pl (P.B.); 2 Department of Biotechnology, Microbiology and Human Nutrition, University of Life Sciences in Lublin, Skromna, 20-950 Lublin, Poland; awasko1@tlen.pl; 3 Department of Crystallography, Faculty of Chemistry, Maria Curie-Skłodowska University, Maria Curie Skłodowska, Square 3, 20-031 Lublin, Poland; daniel.kaminski@poczta.umcs.lublin.pl; 4 Department of Chemistry, University of Life Sciences in Lublin, Skromna, 20-950 Lublin, Poland; iwona.budziak@up.lublin.pl

**Keywords:** black soldier fly, *Hermetia illucens*, chitin isolation, heavy metals

## Abstract

Chitin has become a desirable raw material used in various areas of life. The black soldier fly (*Hermetia illucens*) can be a source of this substance. In the literature, there are many methods of obtaining chitin but there is no one universal method of isolating it. In this publication, we present various procedures for the isolation of chitin from *H. illucens* pupal exuviae. The obtained chitin variants were characterized using different techniques (optical and confocal microscopy, FTIR, XRD, EDX, thermogravimetric analysis). The tested chitin isolated with an efficiency of 5.69–7.95% was the α form with a crystallinity degree of 60% and maximum degradation temperature of 392 °C. Furthermore, we characterized the nickel ion biosorption process on chitin and proposed the mechanism of this process to be ion exchange and complexation. There have been no such studies thus far on the isolation of chitin from *H. illucens* exuviae or on the biosorption of nickel ions on this type of biosorbent. The conducted research can be used to develop the application of chitin as a metal biosorbent that can be obtained with relatively high efficiency and good sorption properties.

## 1. Introduction

It is known that many species of insects play an important role in human life. There are about 2 billion people for whom insects are also part of their traditional diets. Because of the increasing cost and demand for animal protein and the continued growth in human population, insects as a source of food appear to be a great solution for both people and the environment [1]. They contain a high amount of fat, protein, vitamin, fibres and minerals [2]. Other examples of insect use include feed production [1], as a source of chitin and chitosan [3], utilization of organic wastes [4], and biocontrol of pests [5]. They can even participate in the recycling of materials and can be used in the production of soil fertilizer [1]. Chitin is a natural biopolymer whose importance is growing all the time in many industries. It is used for instance in:

- Medicine—in artificial tendons, ligaments, dressings, and wound healing; materials produced from chitin are characterized by haemostatic and antibacterial activity and biodegradability [6,7,8];
- Dietetics—it is used in the production of supplements, food thickeners and pharmaceuticals [9];
- Cosmetics: hair care, skin care, oral care; chitin can be also found in toothpaste, mouthwashes and chewing gum [9,10];
- Others, such as: food packaging industry, additive in paper production, textile industry, adhesives or as a carrier in ion exchange resins [6,10,11,12].

There are many sources from which chitin can be obtained. Examples of frequently used organisms are shrimp, crab, krill and squid [13,14,15,16]. Another source of chitin is sponge skeleton [17,18]. Yet another source of chitin are insects [19,20,21] and among them is *Hermetia* *illucens*, also known as the black soldier fly or black fly [3,22,23].

Many methods of chitin isolation have been developed in recent years [24,25]. Depending on the raw material used to isolate the chitin, there are various undesirable components present in the material that have to be removed. In the case of insects, chitin is present in the chitin–melanin complex [26], in fungi in the chitin–glucan complex [27], in crustaceans, chitin forms a network with proteins where calcium carbonate is deposited [24]. To isolate pure chitin, different chemical (using various reagents) as well as biological (enzymatic deproteinization, fermentation) methods are used. However, there is no one standard method of isolating chitin [24]. Therefore, it is best to experimentally optimize the chitin isolation method based on the raw material used.

One of the possibilities of chitin application is its use as a sorbent in the sorption process [28,29]. For instance, chitin obtained from *H. illucens* has been used as a sorbent of organic dye [30]. Chitin obtained from other sources is a good sorbent for heavy metals [31,32,33]. However, there is a lack of studies on the possibility of using chitin from *H. illucens* in the sorption of heavy metals.

The aim of this work was first to find the best procedure of chitin isolation from *H. illucens* pupal exuviae and usage it as the sorbent for nickel ions (as a representative of bivalent metals) were used.

## 2. Materials and Methods

### 2.1. Insect Material

*H. illucens* was taken from laboratory scale cultivation in the Institute of Agrophysics (Lublin, Poland). Larvae were grown at 26 ± 2 °C with substrate (EUROECO Beszczyński, Chełmża, Poland) humidity of 50–80%. Metamorphosis occurred after three weeks. After metamorphosis the pupal exuviae were used for chitin isolation.

### 2.2. Chemicals

Sodium hydroxide (NaOH) and hydrochloric acid (HCl) (both Merck, Darmstadt, Germany) were used in the demineralization and deproteinization stage. Hydrogen peroxide (H_2_O_2_, 30%) (Standard, Lublin, Poland), potassium manganate (VII) (KMnO_4_) (POCH, Gliwice, Poland) and oxalic acid (C_2_H_2_O_4_) (STANLAB, Lublin, Poland) were used in the depigmentation stage.

Nickel ion (Ni^2+^) solution (1000 mg·dm^−3^) was obtained by the dissolution of nickel nitrate (Ni(NO_3_)_2_·6H_2_O) (POCH, Gliwice, Poland), in redistilled water. nitric acid (HNO_3_, 36%) (POCH, Gliwice, Poland) was used for the pH adjustment of the Ni^2+^ solution.

### 2.3. Chitin Isolation from Hermetia Illucens Exuviae

Our variants of the chitin isolation from *H. illucens* were based on Draczyński’s methodology [34]. To find the best procedure (i.e., the best efficiency and the best quality material), we introduced various modifications in the subsequent stages. Insect pupal exuviae were used, which had been previously cleaned (using ethanol and water), dried (60 °C) and ground. About 10 g of the exuviae were taken for each isolation procedure. First, demineralization was performed with 1 M HCl for 1 h, followed by filtration, and the precipitate was neutralized with NaOH and washed with water. Subsequently, deprotenization was performed with NaOH at 80 °C for 24 h. The precipitate was filtered, neutralized (with HCl), and washed with water. The last stage, i.e., depigmentation, varied depending on the variant. As there are procedures for chitin isolation without the depigmentation stage [35,36], we omitted this stage in one of our variants (variant No. 1). In variant No. 2, depigmentation was carried out by boiling the sample in water at 100 °C for 24 h. In variant No. 3, H_2_O_2_ was used at 80 °C for 2.5 h and for 5 h for variant No. 4. Variant No. 5 was obtained according to the Draczynski procedure using 1% KMnO_4_ at 80 °C for 20 min (as an oxidant) and 4% oxalic acid (as a reducing agent) at 80 °C for 1 h [34]. The final product was obtained by filtration and then neutralized (using NaOH), washed with water and dried at 60 °C for 24 h. The obtained chitin was weighed and the efficiency of the process was calculated according to the formula below.

(1) $$Chitin\;yield=\frac{m}{w}\;\times \;100\%$$

where *m* is the weight of the isolated chitin and *w* is the weight of *Hermetia illucens* exuviae.

The isolations were carried out in three replications for all variants. All the methods described in this work are presented in Table 1.

The isolations were carried out in three replications for all variants.

### 2.4. Nickel Sorption

The isotherms (variant No. 6) were recorded during the sorption of Ni^2+^ ions on chitin isolated according to the procedure described as variant No. 3 (Table 1). This procedure was as follows: the solution of Ni^2+^ ions was adjusted to an initial pH 5. 0.1 g of chitin was mixed with 10 cm^−3^ of Ni^2+^ solution. Five Ni^2+^ ion solutions were used whose concentrations were 10, 20, 40, 80 and 180 mg·dm^−3^. Then, the suspensions of the Ni^2+^ ion solutions and chitin were centrifuged at a speed of 12,000 r.p.m. The concentration of metal was measured in the supernatant using an ICP-OES (Thermo Scientific iCAP Series 6500, Waltham, MA, USA). The entire process was carried out at a constant temperature of 22 ± 1 °C. All the tests were carried out in three repetitions.

### 2.5. Methods of Chitin Characterization and Sorption Effect

The methods described in the following subchapters were used for the characterization of both chitin itself and the chitin with sorbed nickel.

#### 2.5.1. Optical and Confocal Microscopy

Micrographs of the same fragment of the chitin samples and at the same microscope magnification were taken in reflected white light and in the violet light of a laser with a wavelength of λ = 405 nm. The photos were taken with a Nikon Eclipse MA 200 metallographic microscope (Japan) with the Eclipse C1 confocal attachment.

#### 2.5.2. X-Ray Powder Diffraction (XRPD)

The dry organic material was analysed without further grinding, using a powder X-ray diffraction technique with an Empyrean (Malver PANalytical, Cambridge, UK) diffractometer equipped with a Cu anode as the source of CuKα X-ray radiation (λ = 1.5405 Å) and multilayer optics. The input slits were set to ½ and the detector had a background reducing collimator. Both the incident beam path and detector path were corrected with Soller slits (0.02 rad). During measurement, samples were rotated with a speed of 8 r.p.m. All samples were measured over a 2θ range of 4° to 90° with a step size of 0.013° and an exposition time per step of 1 s. All data were collected at 20 °C. The degree of crystallinity was estimated with the use of WaxFit software [37], which is based on the ratio of surface area under curves of crystalline phase to the sum of the area under the curves of the amorphous phases and crystalline phases. All peaks were described (estimated) by Gauss–Cauchy functions. Before fitting, background data was taken into account and smoothed with the Savitzky–Golay method. Diffraction data was compared using ReX software [38] with the α chitin structure from the CIF file presented in the work of Sikorsi et al. [39]. In the fitting procedure, only scale, size of crystallites and background were used.

#### 2.5.3. Thermogravimetric Analysis (TG, DTG, DTA)

Thermogravimetric analysis was performed using a Derivatograph C (Paulik, Paulik and Erdey, MOM, Budapest, Hungary) with corundum crucibles. Previously weighed portions of chitin samples (approximately 10 mg) were analysed in the temperature range of 20–600 °C in an inert nitrogen atmosphere, at a temperature increase rate of 10 °C min^−1^.

#### 2.5.4. Fourier-Transform Infrared Spectroscopy (FTIR)

The FTIR spectra were recorded using a Nicolet 6700 FTIR spectrometer (Thermo Scientific, Madison, WI, USA) equipped with a diamond attenuated total reflectance (ATR) attachment. The FTIR spectra were recorded between 4000 and 400 cm^−1^.

#### 2.5.5. Energy Dispersive X-Ray Spectroscopy (EDX)

The lyophilized samples of chitins (native and enriched with Ni ions) were attached to an aluminium stub using carbon adhesive tape. As is necessary in EDX analysis, the samples were pressed to obtain a flat and smooth surface. A FEI Quanta 3D FEG scanning microscope was used. EDX spectra were obtained from five different areas of each sample. To increase the representativeness of measurements, multiple measurements were carried out. Elemental composition of the samples was calculated with the use of EDAX Genesis software.

### 2.6. Statistical Analysis

The values from all tests were the mean values of three separate experiments ± standard deviation. Tukey’s HSD test and Student t-test (STATISTICA 8.0, StatSoft, Inc., Tulsa, OK, USA) was used for the determination of statistical differences with the significance denoted at *p* < 0.05.

## 3. Results

### 3.1. Chitin Isolation from H. illucens Pupal Exuviae

Information concerning chitin isolation efficiency is presented in Table 2. It can be seen that the less aggressive last stage of chitin isolation (depigmentation) gave the higher isolation yield. The pigment was destroyed using 9% hydrogen peroxide (H_2_O_2_) (variant Nos. 3 and 4) while in variant No. 5 we used potassium permanganate (KMnO_4_). For variant No. 2, the sample was boiled in water for 24 h to remove the pigment. Generally, we observed that type of oxidant, time and temperature are the likely cause of chitin degradation in this step of the procedure. The best isolation method was selected based on the yield and the purified material properties (the biggest pore-like structure which should favour the sorption process and the highest temperature decomposition which should favour a wide range of applications).

### 3.2. Optical and Confocal Microscopy

**Figure 1:**
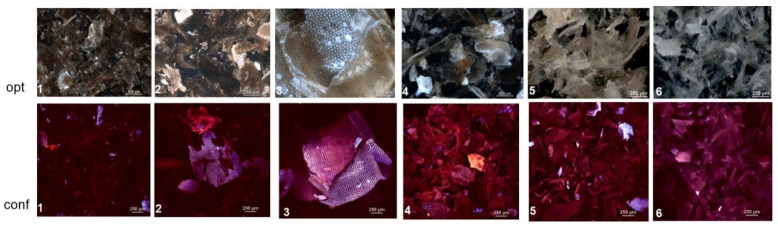
Examples of photographs of the investigated samples obtained by optical microscope (**top row**) and confocal microscope (**bottom row**). The numbers in the bottom left corners correspond to the numbers of the variants (Table 1).

Examples of photos made by both of the microscopes are presented in Figure 1.

It is difficult to notice significant changes in the optical microscope images for individual variants. Generally, it can be stated that the chitin surface resembles a honeycomb or porous structure, especially in variant 3.

Based on the confocal photos it can be stated that variant No. 1 showed fluorescence of only some objects due to the absorption of monochromatic light and a colour shift in the direction of longer wavelengths (blue and red), and most of the chitin sample glowed with reflected light (violet); variant Nos. 2 and 6 were similar. Variant 3 showed a slightly higher fluorescence, although most of the sample was still illuminated with reflected (violet) light. Next, variant No. 4 showed strong fluorescence of the objects, while the variant No. 5 had lower fluorescence than variant No. 4.

### 3.3. X-ray Powder Diffraction (XRPD)

**Figure 2:**
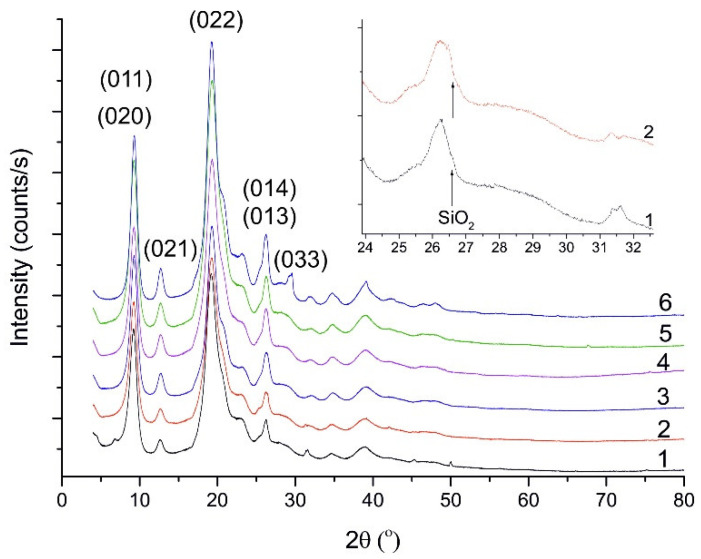
The diffraction pattern of α chitin for all isolation variants and after Ni^2+^ sorption. The presented data corresponds to the six variants from Table 1. The background was used to estimate the degree of crystallinity. The inset shows a trace of SiO_2_ in samples 1 and 2.

Figure 2 shows the X-ray powder diffraction patterns of all samples. Comparison of the experimental data to records from powder diffraction databases and with the literature [39] indicates the data are related to the alpha chitin. The crystallite size is ~80 nm in all cases as indicated by Rietveld analysis based on the crystallographic structural data provided by Sikorksi at al. [39].

The degree of crystallinity of the samples was estimated to be around 60%. Summarising this part of the results it can be stated that the preparation method has no effect on the chitin crystallite.

Trace silica (quarts-α SiO_2_) amounts were found in two variants (Nos. 1 and 2), as it is indicated by weak reflection at 2θ = 26.6° (Figure 2, insets).

### 3.4. Thermogravimetric Analysis (TG, DTG, DTA)

**Figure 3:**
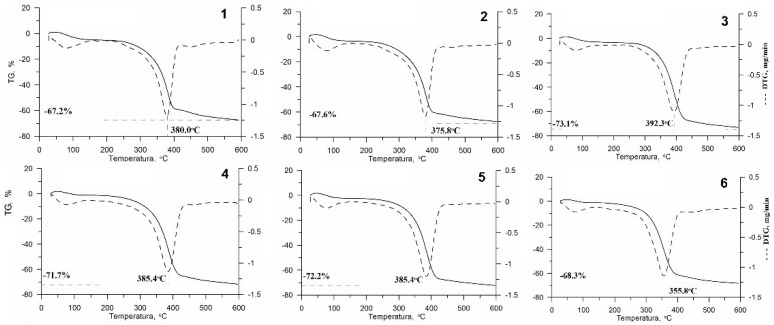
Thermogravimetric analysis (TG) (solid line) and Derivative Thermogravimetry (DTG) (dotted line) curves of chitin from black soldier fly obtained by the isolation variants and after the Ni^2+^ sorption process. Numbers are related to the variants presented in Table 1.

All the curves obtained from thermogravimetric measurements are presented in Figure 3 (Thermogravimetric—TG and Derivative Thermogravimetry—DTG) and 4 Differential Thermal Analysis curves (DTA). On the basis of these, it was found that the chitin mass changes are related to two endothermic transitions. The evaporation of water at temperatures in the range of 80–100 °C was the first (inset 1). The second was the decomposition of chitin itself at temperatures in the range of 356–392 °C, depending on the sample (Figure 3). The loss of mass during decomposition for individual samples was determined from the TG curve and it was between 67.2% and 73.1%, depending on the sample.

The highest temperature of chitin decomposition and the highest weight loss occurred in variant No. 3 (i.e., chitin treated with H_2_O_2_ for 2.5 h), the lowest in the variant No. 6 (i.e., after Ni^2+^ ions sorption).

**Figure 4:**
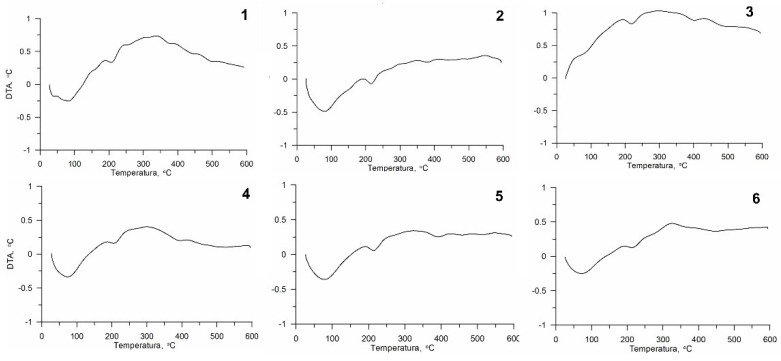
Differential Thermal Analysis (DTA) curves of chitin from black soldier fly for all variants of chitin isolation and after the Ni^2+^ sorption process. Numbers are related to the variants presented in Table 1.

There were no clearly visible peaks in the DTA curves for any of the samples tested (Figure 4). However, it can be seen that at about 80 °C there was an endothermic transformation (for all samples except variant No. 3), and this reaction was probably caused by the evaporation of water; and another at 300 °C associated with the exothermic decomposition of chitin.

### 3.5. Fourier-Transform Infrared Spectroscopy (FTIR)

**Figure 5:**
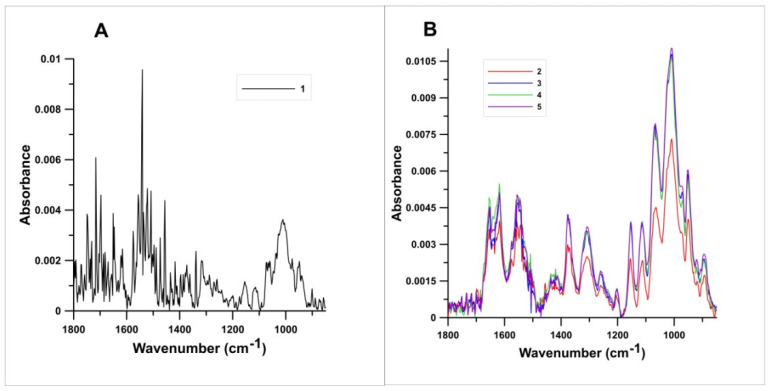
FTIR spectra for different variants of chitin isolation. Numbers are related to the variants presented in Table 1. Because the spectrum of variant No. 1 was completely different than the others (**B**) it was presented as a separate spectrum (**A**).

The results of infrared spectroscopy on the different variants of chitin are represented in Figure 5A,B, respectively. Variants Nos. 2–5 showed no significant differences between the FTIR spectra (Figure 5B), while additional peaks are visible in the FTIR spectrum of the sample in which the depigmentation process was not performed (variant No. 1) (Figure 5A). The sample without the depigmentation decolourization step presented much sharper peaks then samples isolated with the depigmentation steps. The spectra of the samples isolated with variant Nos. 2–5 have two characteristic bands, one at 1620 cm^−1^ and another at 1652 cm^−1^. These bands are attributed to the amide I vibrations, especially C=O secondary amide stretching vibration. Additionally, the band at 1552 cm^−1^ correspond to a N–H bending, C–N stretching typically characteristic for amide II. The sharp band at 1307 cm^−1^ corresponds to a –CH_2_-group (amide III), due to the formation of CO–NH components of protein. The other FTIR spectrum bands were observed as the following: 1377 cm^−1^ (C–H bend, –CH_3_ symmetric deformation), 1557 cm^−1^ (N–H deformation of amine II), 1067 cm^−1^ (C–O–C asymmetric stretching in phase ring), 1008 cm^−1^ (C–O asymmetric stretching in phase ring) and 951 cm^−1^ (−CH_3_ wagging).

### 3.6. Sorption of Nickel Ions

**Figure 6:**
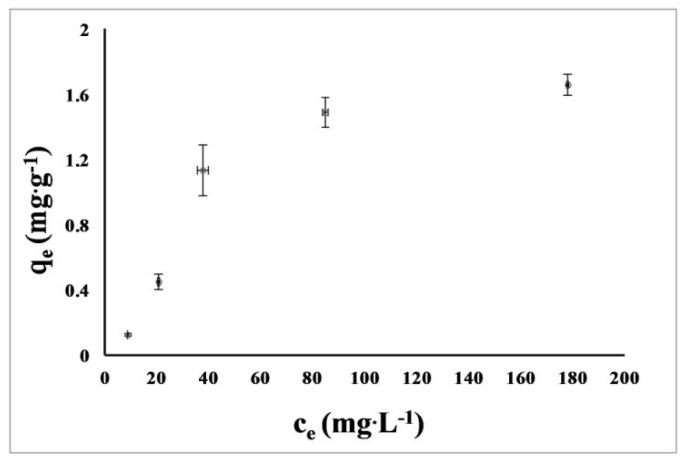
Isotherm curve for the adsorption of Ni^2+^ onto chitin isolated from *H. illucens* (means (*n* = 3) ± SD (bars)) by procedure No. 3 (see Table 1) where c_e_ is the equilibrium Ni^2+^ concentration; q_e_ is the amount of Ni^2+^ adsorbed at equilibrium.

The isotherm showed a gradual increase in adsorption with increasing metal concentration, after which equilibrium was reached and the sorbent surface was saturated with metal (Figure 6). The obtained adsorption isotherm of Ni onto chitin was tested on two adsorption models (Langmuir and Freundlich). The parameters of each model are presented in Table 3. Analysis of these data showed that the better fit was obtained for the Freundlich isotherm.

### 3.7. Energy Dispersive X-Ray Spectroscopy (EDX)

EDX analysis was performed mainly to confirm nickel ion sorption on chitin and study the biosorption mechanism. Therefore, for this purpose the chitin sample depigmented with H_2_O_2_ for 2.5 h (variant No. 3) and the same sample after Ni^2+^ sorption (variant No. 6) were used. Table 4 presents percentage content of individual elements. The analysed chitin samples consisted of about 60% carbon, about 25–28% oxygen, and about 9–12% nitrogen. Other elements included in the chitin were sodium and chlorine, possibly in the form of contaminating NaCl and traces of Al, Si and Ca. The content of Ni on the surface of the tested chitin after adsorption was 0.23%. A decrease in the content of elements (Na, Al, Si, S and Ca) was observed after the sorption. There were no statistically significant differences between variants Nos. 3 and 6, except for chlorine and elements whose determined amount was below the lower limit of quantification.

## 4. Discussion

### 4.1. Chitin Isolation from H. illucens Pupal Exuviae

The chitin isolation yield for individual variants ranged from 5.69% to 7.97% and depended on the reagent used in the depigmentation step—the stronger the reagent, the lower the isolation yield. The literature data on the isolation yield varies considerably, e.g., the isolation yield of chitin from silkworm was 2.59–4.23% [40], and from bees 18% [34], or even 19–36.8% [41]. However, these are results for chitin isolated from other sources, and the organisms differ in their chitin content [22]. Chitin was isolated from *Hermetia illucens* with a yield of about 20%, so much higher than in this study [22,25,42]. The first difference between our work and the cited studies is the source of chitin isolation—in our case it was pupal exuviae, while the other authors used dead flies. A recent study by Shin et al. confirm that chitin yield from different stages of the life cycle of the insect (larvae, pupa, and adults) showed values between 3.9% and 14.2% [43]. Another difference between the presented work and the results from the literature is the use of different concentrations of the same reagents for a different period of time and at a different temperature. Previously, chitin was isolated from *Hermetia illucens* using hydrochloric acid (5%) (at room temperature, for 2 h) and sodium hydroxide (5%) (at 100 °C, for 3 h), then the obtained precipitate was washed with ethanol, and its residues were removed with chloroform [22]. On the other hand, Hahn et al. used three different methods to measure chitin content in various insects (also *Hermetia illucens*): acid detergent fibre (ADF), acid detergent lignin (ADL), a combination of both (ADF-ADL) and acetyl group measurement. The authors concluded that the best is ADF-ADL, taking into account the statistical analysis of the results, equipment requirements, accuracy and universality of the method [42]. Brigode et al. compared the conventional method of chitin isolation from black soldier fly using acid and base (1 M HCl at 100 °C for 30 min and 1 M NaOH for 24 h) with the ADF, ADL and ADF-ADL methods [25]. The yield of chitin obtained by the conventional and ADF-ADL methods was similar, higher results were obtained for the ADF method (3–10% higher) [25].

Abidin et al. conducted an extensive review of the literature on chitin and chitosan. They compared the methods of chemical isolation and raw materials used. Insects have been given particular attention as an alternative source to commonly used crustaceans [20]. It is difficult to choose one single method of chitin isolation that could be used for any raw material. When selecting the isolation method, it is most important not to degrade the chitin during its purification [20]. It was also noticed that the method of obtaining chitin affects the properties of the sample, so the choice of method should take into account the future use of the chitin [20]. Based on data collected by previous researchers [20], results from our work indicate that the isolation procedures (especially variant No. 3) used in this study can be used for the extraction of chitin from pupal exuviae *H. illucens.*

### 4.2. Optical and Confocal Microscopy

The obtained chitin samples were visually assessed with the use of optical and confocal microscopes. All samples tested had a regular surface consisting of hexagonal units. Similar results had been obtained previously using an SEM microscope [3,25]. Chitin does not have the ability of autofluorescence [44]. Chitin studies have been conducted using a confocal microscope, but the use of an appropriate reagent (e.g., fluostain) is required [45]. A new technique that enables chitin testing without sample preparation is nonlinear microscopy [46]. In this publication, the performed microscopic assessment of the samples does not provide information on the purity of the obtained materials and does not differentiate the samples but we observed a number of pores in the chitin surface (variant No 3). Same authors [10] have stated that this structure increased the chitin’s ability to absorb metal ions. For this reason, this variant was chosen for the sorption of nickel ions. The strong fluorescence of the variant No. 4 is probably due to the use of a strong oxidant (H_2_O_2_) for a sufficiently long time (5 h) compared to the variant No. 5, where the fluorescence was weaker as KMnO_4_ was used for depigmentation for much shorter time (20 min). It is difficult to notice significant differences in the images of the optical microscope between variant No. 6 (after sorption) and variant No. 3 (without sorption). Variant No. 6 showed weak fluorescence due to the absorption of monochromatic light; the practical total of the chitin sample glowed with reflected light (violet), while the fluorescence of variant No. 3 was slightly higher.

### 4.3. X-Ray Powder Diffraction (XRPD)

As mentioned above, chitin crystallite size was ~80 nm in all investigated isolation variants. A recent study confirmed these data concerning crystallite size in different forms of chitin [47]. This should be expected because the source of the chitin was the same in all cases and the isolation procedures used should not affect the crystallographic pattern.

Sorption of Ni^2+^ on chitin isolated in variant No. 3 leads to more sharp reflections in the XRPD diffraction pattern. Only this sample has a clear and sharp (0 3 3) reflection.

The degree of chitin crystallinity was similar in all chitin isolation variants and was around 60%. In the literature, the values for this parameter are very wide. Kaya et al. [48] reported that chitin crystallinity was in the range 40–80%. However it is also possible to find lower values, for instance: 25.2% from *H. illucens* pupae [49], 35% from pupae and 24.9% for imago chitin [3] or 38.82% from *H. illucens* prepupae [50]. The degree of crystallinity is strongly influenced materials sorption properties [51]. However, it should be remembered that the longer the grinding time of the sample, the smaller degree of crystallinity [52]. Therefore, it is more important to pay attention to the dependence of the degree of deacetylation (related to the presence of amino groups) and the sorption capacity [53,54,55].

### 4.4. Thermogravimetric Analysis (TG, DTG, DTA)

The decomposition of the tested chitin obtained with various isolation variants took place in two stages, which is consistent with the literature data [49,56], although there are also older articles describing a single-stage decomposition process [57,58,59]. The temperatures for the individual stages of different chitin variants were consistent with the literature data [49,60]. The decomposition temperature of chitin was in the range of 356–392 °C, and, for example, for the commercial chitin DTGmax it is 386 °C [48].

The applied methods of isolation affected the temperatures of decompositions and therefore it can be concluded that it influenced the composition of the obtained chitin. Although all tested samples were thermally stable, there were clear differences—up to 16 °C. The DTG max values (375.8–392.3 °C), which are the maximum decomposition temperatures, were similar for all samples of isolated chitin (variant Nos. 1–5). The highest decomposition temperature was obtained for variant No. 3 (i.e., chitin treated with H_2_O_2_ for 2.5 h), which makes this procedure the most favourable because the higher the decomposition temperature, the wider the possibility of chitin usage. In previous studies, it has been seen that the DTG max value of alpha chitin varied between 350 and 400 °C [61,62].

The mass loss for the tested chitins from the exuviae was 67.2–73.1%, these values are lower compared to the literature for the same material (95%) [49]. Purkayastha et al. studied chitin isolated from *Hermetia illucens*, but the DTA curves showed clear exothermic peaks in the range 339–430 °C [5]. The most comparable result was obtained for variant No. 3.

### 4.5. Fourier-Transform Infrared Spectroscopy (FTIR)

Infrared spectroscopy is one of the classical methods for chitin identification, FTIR spectra provides detailed information on the presence of amide and amine groups and, therefore, is one of the most commonly used methods for the characterization of the degree of acetylation in chitin samples [63]. FTIR has been used to determine chitin allomorphs (alpha-, beta- and gamma-) [64] and to analyse the demineralization, deproteinization, and decolorization processes when isolating chitin from raw materials [65]. It is known that alpha chitin is found in insects [48]. In the FTIR spectra of the chitin isolated in this study, the amide I band was split at 1620 and 1652 cm^−1^, which indicates that the chitin from *H. illucens* was in the alpha form, which has already been confirmed by the XRPD. These peaks presented in the samples regardless of the isolation method. Moreover, the chitin from *H. illucens* displayed the amide II band at wavenumber 1552 cm^−1^ just as chitins from Orthopthera species examined by Kaya et al. [64]. The band 1307 cm^−1^ was assigned to CON–H deformation and to the –CH_2_– group (amide III). In addition, the wavenumbers at 1067 and 1008 cm^−1^ were observed to correspond to the asymmetric and symmetric stretching vibration of the C–O–C group. Similar FTIR results have been obtained for alpha chitin isolated from various insect species [47].

The spectrum of chitin with sorbed Ni^2+^ ions was very similar to those presented in Figure 5B, i.e., for variants Nos. 2–5 and it was not presented in order not to reduce the clarity of the figure. However, one observation of this spectrum analysis seems to be important: a slight shift of bands 3369 and 2910 cm^−1^ occurred towards longer lengths (3380 and 2920 cm^−1^, respectively) after metal sorption. The bands correspond to the stretching vibrations of the –OH groups, hence it can be concluded that they participate in the adsorption of the metal.

Sorption was probably at least partly based on complexation involving –OH groups on the surface of the tested sorbent (chitin).

### 4.6. Sorption of Nickel Ions

Studies on the sorption of various heavy metals on native and modified chitin are available in the literature. The equilibrium sorption data fit both the Langmuir and Freudlich isotherms [66,67,68,69]. It is influenced by the type of metal used for sorption, but it also seems that chitin isolated from various sources also has different properties, which also translates into metal sorption. In our study, the chitin adsorbed 1.66 mg·g^−1^ of nickel ions at pH 5.0 and nickel concentration 178 mg·dm^−3^. It is difficult to compare the data on the sorption of metals on chitin due to the different method of conducting the adsorption process (using different weights of sorbent, different volumes of added metal solutions, different procedures and process durations). Karthik et al. studied commercial chitin modified with polypyrrole to remove Pb (II) and Cd (II) ions. The maximum metal removal capacity was 98.20% for Pb (initial 2.79 mg·dm^−3^ and final 0.05 mg·dm^−3^ concentration) and 95.77% for Cd (initial 2.84 mg·dm^−3^ and final 0.12 mg·dm^−3^). The obtained isotherms showed similarity to the Freudlich isotherm [68]. Forutan et al. used pink shrimp chitin as a sorbent for lead ions. The highest ion removal capacity was 99.7%, with an initial metal concentration of 20 ppm. The designated isotherm showed a better fit to the Freudlich isotherm [67]. Xiang et al. studied the adsorption of Cd(II) ions on chitin. The maximum absorbed amount of metal was 93.9 mg·g^−1^, and the obtained isotherm data showed similarity to the Langmuir equation [69]. Chui et al. removed metals (Cu, Cr and Ni) from aqueous solutions using chitin (derived from shrimp) packed in small columns using the Solid Phase Extraction (SFE) technique. The removal capacity of Cu and Cr was 95% and 96% respectively, while for Ni it was much lower and amounted to 44–70% (at the initial metal concentrations of 20–100 mg.dm^3−^) [70]. Yazidi et al. used ultrasonic modified chitin (from shrimp) for multi-component adsorption of Ni, Co, and Methylene Blue. The equilibrium concentration for Ni, Co and Methylene Blue was 58 mg·g^−1^, 37 mg·g^−1^ and 6 mg·g^−1^, respectively. To determine the isotherms, 50 mL of solution containing three components with an initial concentration of up to 650 mg.dm^−3^ and 5 g of adsorbent were used [71]. Although no research has been done on metal sorption on chitin from *H. illucens*, the larvae of this insect have been used to treat municipal sewage sludge in China where heavy metals are a major problem. *H. illucens* showed great tolerance to heavy metals (including Ni, Cu, Hg, Cd); the examined metals did not significantly affect the metamorphosis and life of insects, but they slightly limited the increase in mass [72].

In order to determine the mechanism of Ni sorption on chitin, analyses were carried out using other techniques. It seems that the two sorption mechanisms presented by Kołodyńska et al. [73], i.e., ion exchange and complexation, should be taken into account. The EDX results (Table 4) showed the presence of Al and Ca before Ni sorption and these metals were not visible after sorption. The amount of Na decreased after Ni sorption. These decreases may be caused by ion exchange. Although EDX measured only a limited surface and limited depth of the material, the averaged results from multiplied measurements from different individual points on a given sample (Table 4) confirmed the above reasoning.

Information about the parallel second mechanism of sorption, i.e., complexation can be derived from FTIR results. The evidence for complexation mechanism is the shift of bands corresponding to stretching vibrations of the –OH groups. Similar conclusions regarding the sorption mechanism have already been reported [68,72]. Karthik et al. proposed that the sorption of Pb (II) and Cd (II) probably occurs through ion exchange and complexation [68]. According to other authors, the complexation of Cd (II) ions occurs through an acetylamino (–CONH–) and a hydroxyl (–OH) group [72].

### 4.7. Energy Dispersive X-Ray Spectroscopy (EDX)

The EDX technique made it possible to compare the content of individual elements before and after sorption, and then to confirm the sorption process and make conclusions about its mechanism. In the variant No. 6, the presence of Ni ions on chitin was found, which confirms the biosorption of the tested metal. Comparing variant No. 3 and variant No. 6, a decrease in Na content can be seen. It can be assumed that this decrease may be due to ion exchange. Likewise, Acheampong et al. observed changes in the content of K and Mg in agricultural materials after the sorption process, which were replaced by the tested Cu ions [74].

## 5. Conclusions

In this study, we extracted chitin from *Hermetia illucens* pupal exuviae using different chemical methods. We obtained a chitin yield of 5.69–7.95% which is comparable with other insect-specific studies. Taking into account the isolation yield and the chitin properties determined by many methods (optical and confocal microscopy, FTIR, XRD, EDX, thermogravimetric analysis we selected the best procedure of chitin isolation consisting of the following stages: (i) demineralization (1 M HCl at 22 °C for 1 h. Then neutralization with NaOH to obtain neutral pH. Rinsing by distilled water), (ii) deproteinization (1 M NaOH at 80 °C for 24 h. Then neutralization with HCl to obtain neutral pH. Rinsing with distilled water), (iii) depigmentation (9% H_2_O_2_ at 80 °C for 2.5 h). The longer use of H_2_O_2_ and more aggressive agent (KMnO_4_) at the depigmentation stage resulted in a decrease in isolation yield without increasing the positive properties of chitin. The chitin extracted from *H. illucens* is in α-form, with a crystallinity degree of 60%, and maximum degradation temperature of ~392 °C. Furthermore, for the first time, we analysed and described the biosorption process of nickel ions on chitin from *H. illucens*. Sorption of Ni^2+^ ions on our chitin was comparable to the sorption on chitin of a different origin. However, it is difficult to make a detailed comparison because this type of research on nickel sorption on chitin obtained from *H. illucens* could not be found. We found that in this study, the mechanisms of ion exchange and complexation are responsible for the sorption process.

## Figures and Tables

**Table 1:** Variants of the isolation methods presented in this work. Variant numbers specified in this table are used in the entire text.

Variant No.		Demineralization Stage	Deproteinization Stage	Depigmentation Stage
chitin isolation	1	1 M HCl to obtain acidic reaction at 22 °C for 1 h. Then NaOH to obtain neutral reaction. Rinsing by distilled water	1 M NaOH at 80 °C to obtain alkaline reaction for 24 h. Then HCl to obtain neutral reaction. Rinsing by distilled water.	Without the stage of depigmentation
	2			Water at 100 °C for 24 h
	3			9% H_2_O_2_ at 80 °C for 2.5 h
	4			9% H_2_O_2_ at 80 °C for 5 h
	5			1% KMnO_4_ at 80 °C for 20 min. Then oxalic acid (4% C_2_H_2_O_4_) to reduce the excess of KMnO_4_. Then NaOH to precipitate the manganese. Rinsing by distilled water
	6	Sorption of Ni^2+^ on chitin isolated in variant 3 procedure		

**Table 2:** The efficiency of chitin isolation from *H. illucens* pupal exuviae depending on the isolation variant (means and SD, *n* = 3). The variants are described in Table 1. The same letter means that there were no statistically significant differences (Tukey’s HSD test; *p* < 0.05).

Variant Number	1	2	3	4	5
Yield (%)	7.95 ± 0.20 ^b^	7.97 ± 0.10 ^b^	7.01 ± 0.12 ^c^	5.98 ± 0.08 ^a^	5.69 ± 0.28 ^a^

**Table 3:** Parameters of isotherms obtained during the sorption of Ni^2+^ on chitin.

Langmuir			q_e exp_ [mg·g^−1^]	Freundlich		
K_L_	q_m_ [mg·g^−1^]	R^2^		K	1/n	R^2^
0.008	3.086	0.620	1.662	0.010	1.179	0.860

q_m_—the maximal theoretical adsorbed amount (sorption capacity), K_L_—the Langmuir constant—the quasi Gaussian energetic heterogeneity of the adsorption system, R—determination coefficient, q_e exp_—the maximal experimental amount adsorbed at equilibrium time, K, n—empirical constants indicative of sorption capacity and sorption intensity.

**Table 4:** The content of individual elements in the sample of chitin variant 3 and samples of chitin variant 3 with sorbed of Ni^2+^ determined by EDX (means and SD, *n* = 5).

	Variant No. 3	Variant No. 6
%C	61.56 ± 7.40	62.71 ± 3.35
%N	11.60 ± 3.64	10.90 ± 1.69
%O	25.88 ± 4.93	25.28 ± 3.19
%Na	0.37 ± 0.19	0.18 ± 0.12
%Al	0.18 ± 0.15	-
%Si	0.11 ± 0.10	-
%S	0.33	0.07 ± 0.04
%Cl	0.33 ± 0.16	0.64 ± 0.19
%Ca	0.07	-
%Zn	-	-
%Cd	-	-
%Ni	-	0.23 ± 0.06
%Pb	-	-

## Data Availability

Not applicable.
